# A ketogenic diet improves the prognosis in a mouse model of peritoneal dissemination without tumor regression

**DOI:** 10.3164/jcbn.18-103

**Published:** 2019-03-07

**Authors:** Eiji Kasumi, Norifumi Sato

**Affiliations:** 1 EN Otsuka Pharmaceutical Co., Ltd., R&D Laboratories, 4-3-5 Nimaibashi, Hanamaki, Iwate 025-0312, Japan

**Keywords:** ketogenic diet, peritoneal dissemination, ascites, VEGF-A, β-OHB

## Abstract

Peritoneal dissemination describes a state where tumor cells spread to the surface of the peritoneum and become engrafted. Peritoneal dissemination reduces the quality of life and prognosis of cancer patients. Currently, there are few effective therapies or preventative treatments for peritoneal dissemination. The aim of this study was to evaluate a ketogenic diet, characterized by high fat, moderate protein and low carbohydrate content, as a novel therapy in a mouse model of peritoneal dissemination. BALB/c mice were intraperitoneally inoculated with colon 26, a murine colon adenocarcinoma cell line, to induce experimental peritoneal dissemination. After tumor inoculation, mice were fed a regular or ketogenic diet. A longer survival time and better health status score, related to improved behavior, was observed in the ketogenic diet group compared with the regular diet group. In addition, the weight of ascites was significantly smaller and the anemia symptoms, number of red blood cell, hemoglobin and hematocrit, were improved in the ketogenic diet group compared with the regular diet group. However, the tumor weight was not significantly smaller in the ketogenic diet group compared with the regular diet group. These data suggest that a ketogenic diet might be a potential preventive therapy for peritoneal dissemination.

## Introduction

Peritoneal carcinomatosis represents a severe form of metastatic cancer progression, which is observed in 10–35% of colorectal cancer patients and 50% of gastric cancer patients.^(1)^ The resulting peritoneal dissemination is the most common and severe clinical symptom, leading to a poor prognosis.^(2)^ Peritoneal dissemination is observed in about 10% of colorectal cancer patients and it causes ascites, extreme fatigue, abdominal discomfort, abdominal pain, and bowel obstruction, with a 5-year survival rate less than 10%.^(3,4)^ Of note, patients with bowel obstruction caused by peritoneal dissemination originating from colorectal cancer as the primary lesion have a worse prognosis with a median survival of approximately 3 months, even after surgical treatment.^(5)^

Recently, it was reported that the overall median survival of peritoneal dissemination patients who received hyperthermic intraperitoneal chemotherapy, which combines cytoreductive surgery and heated chemotherapy in the peritoneal cavity, was 19.2 months and the 3-year and 5-year survival rates were 39% and 19%, respectively.^(6)^ Although the prognosis of patients with peritoneal dissemination derived from colorectal cancer has improved because of the development of new therapies, more effective therapies are still required.

A ketogenic diet, consisting of high fat, moderate protein, and low carbohydrate levels, restricts glucose and generates ketone bodies as an energy source, and has been used to treat childhood epilepsy.^(7)^ Carbohydrate deficiency induces the compensatory synthesis of acetyl-CoA in the liver via fatty acid oxidation and then excess acetyl-CoA is converted to β-hydroxybutyrate (β-OHB) and acetoacetate in the liver mitochondria. Cancer cells mostly generate adenosine triphosphate (ATP) through converting glucose to lactate by anaerobic glycolysis in the presence of oxygen instead of mitochondrial oxidative phosphorylation as seen in normal cells, which is termed “the Warburg effect”. Although anaerobic glycolysis is less efficient than oxidative phosphorylation for generating ATP, the rate of ATP production from glycolysis is about 100 times faster than that from oxidative phosphorylation.^(8)^ Moreover, anaerobic glycolysis supplies cancer cells with intermediate metabolites, nucleic acids, fatty acids, and amino acids, which are required for proliferation.^(9)^ There are some reports that dietary carbohydrate restriction or glucose analogs which don’t undergo glycolysis can actually inhibit tumor growth.^(10–12)^ Thus, glucose has a crucial role in the survival and proliferation of cancer cells through anaerobic glycolysis.

Focusing on the above cancer metabolism mechanisms, we hypothesized that a ketogenic diet will improve the disease condition of peritoneal dissemination by supplying ketone bodies as an energy source and reducing the glucose supply to cancer cells. Additionally, because ketone bodies inhibit histone deacetylase,^(13)^ they might exert a similar effect to that of Vorinostat,^(14)^ an anticancer agent. Although previous studies demonstrated the efficacy of a ketogenic diet on cancers in animal experiments or small-scale clinical trials, its use has not been reported for refractory cancers, in particular peritoneal dissemination.

Here, we demonstrate that a ketogenic diet prolonged the survival rate of a mouse peritoneal dissemination model. Unexpectedly, the ketogenic diet did not have an anti-cancer effect against peritoneal tumors *in vivo* despite lowering the glucose level and elevating ketone body levels in the blood. Furthermore, ketone bodies had no effect on colon cancer cell growth *in vitro*. Although we did not confirm our hypothesis, we found that a ketogenic diet dramatically reduced ascites retention associated with peritoneal dissemination. Our findings indicate that a ketogenic diet might be a novel potential therapy for peritoneal dissemination.

## Materials and Methods

### Animals

Male, 5-week-old BALB/c mice (CLEA Japan, Inc., Tokyo, Japan) were housed in wire cages under a controlled temperature (23 ± 3°C) and humidity conditions (50 ± 20%), with a 12-h light-dark cycle. The mice were fed commercial laboratory chow, and were allowed drinking water *ad libitum* for about one week before the experiments began. All animal experiments conformed to the guidelines for the care and use of laboratory animals established by the Animal Use and Care Committee of EN Otsuka Pharmaceutical Co., Ltd.

### Cell culture and experimental peritoneal dissemination mouse model

The colon 26 cell line was purchased from RIKEN BioResource Center (Tsukuba, Japan). Colon 26 cells were cultured in RPMI-1640 medium supplemented with 10% fetal bovine serum, 2 mM l-glutamine, 100 U/ml penicillin, and 100 µg/ml streptomycin under 5% CO_2_ in air at 37°C. Cultured colon 26 cells were harvested with Accutase (Nacalai Tesque, Inc., Kyoto, Japan) and prepared as a cell suspension at a concentration of 5 × 10^6^ cells/ml in phosphate-buffered saline (PBS). Mice were intraperitoneally inoculated with a 0.1 ml cell suspension using a 26 G needle.

### Experimental design

After tumor inoculation, mice were randomly divided into two groups and fed a ketogenic diet or regular diet (Table 1) *ad libitum*. Survival analysis was performed and the health scores of mice were recorded according to the following parameters: appearance 0–2, natural behavior 0–3, provoked behavior 0–3, and body condition 1–5 in accordance with the method of Paster *et al.*^(15)^ Mice with a total score of 3 or less were euthanized humanely and their survival time was recorded.

To investigate the effect of a ketogenic diet on the disease conditions of the peritoneal dissemination model in detail, tumor cell bearing mice were fed the experimental diet for another 15 days as a separate experiment. Finally, all mice were euthanized and tumors, ascites fluid, and tissues were collected and weighed.

### Blood measurements

On the day of necropsy, blood samples were obtained from the vena cava under isoflurane anesthesia. For clinical chemistry analyses, all blood samples were centrifuged at 1,500 × *g* for 15 min at 4°C, after which the serum was collected. β-OHB was measured using Precision Xceed (Abbott Japan Co., Ltd., Tokyo, Japan). A hematological analyzer XT-1800iV (SYSMEX Corp., Hyogo, Japan) and a clinical chemistry analyzer (Fuji Drichem 3500V, FUJIFILM Medical Co. Ltd., Tokyo, Japan) were used for hematological assessment and serum chemistry respectively, according to manufacturer’s instructions.

### Quantification of vascular endothelial growth factor A (VEGF-A)

VEGF-A in serum and ascites fluid was quantified by a Quantikine kit (R&D Systems, Inc, Minneapolis, MN) according to the manufacturer’s instructions.

### RT-PCR and mRNA quantification

For RNA preparation, Isogen (Wako Pure Chemical Industries, Ltd., Osaka, Japan) was used according to the manufacturer’s instructions. cDNA synthesis was performed using Prime Script Transcriptase (Takara Bio, Inc., Shiga, Japan). For semi-quantitative RT-PCR, gene-specific fragments were obtained by linear phase PCR amplification, and normalized by β-actin level. The specific primer pairs were: VEGF-A 5'-AGACACACCCACCCACATACA-3' (forward), 5'-ACATCCTCCTCCCAACACAAG-3' (reverse); hypoxia-inducible factor (Hif)-1α 5'-GGGTACAAGAAACCACCCAT-3' (forward), 5'-GAGGCTGTGTCGACTGAGAA-3' (reverse); forkhead box O 3A (FoxO3A) 5'-CTGGGGGAACCTGTCCTATG-3' (forward), 5'-CTTCATGCGCGTTCAGAATGA-3' (reverse); β-actin 5'-AGTGTGACGTTGACATCCGT-3' (forward), 5'-TGCTAGGAG CCAGAGCAGTA-3' (reverse).

### WST-8 assay

For cell counting, we used a WST-8 assay kit (Dojindo, Kumamoto, Japan) according to the manufacturer’s instructions. Cells were cultured with β-OHB (Sigma, St. Louis, MO) at each concentration for 48 h, and then a culture medium containing WST-8 solution was added. After a 1-h incubation at 37°C, the absorbance in each well was measured at wavelengths of 450 nm (test wavelength) and 700 nm (reference wavelength) with a Microplate Reader SH-1000 (CORONA, Niigata, Japan).

### Carboxyfluorescein diacetate succinimidyl ester (CFSE) dilution assay

Measurement of cell division was performed by CFSE dilution assay as previously described.^(16)^ Briefly, colon 26 cells were incubated with CFSE (Wako Pure Chemical Industries, Ltd.) in PBS at 37°C for 1 h to apply the fluorescence label and then plated into 6-well plates at a density of 2 × 10^5^ cells/ml with β-OHB. After 2 days of incubation, the harvested cells were excited with a laser at a wavelength of 488 nm and the fluorescence intensity of the CFSE per the cell was quantified by FACSVerse flow cytometer (BD Biosciences, San Diego, CA).

### Statistical analysis

The results are expressed as the means ± SD. Statistical analysis was performed by Student’s *t* test or Welch’s *t* test based on the result of an *F* test. The Kaplan-Meier method was used to analyze the survival rate and the log-rank test was applied to compare the survival curve. *P* values less than 0.05 were considered statistically significant.

## Results

### Ketogenic diet prolongs survival and improves the health condition score in mice with peritoneal dissemination

**Fig. 1:**
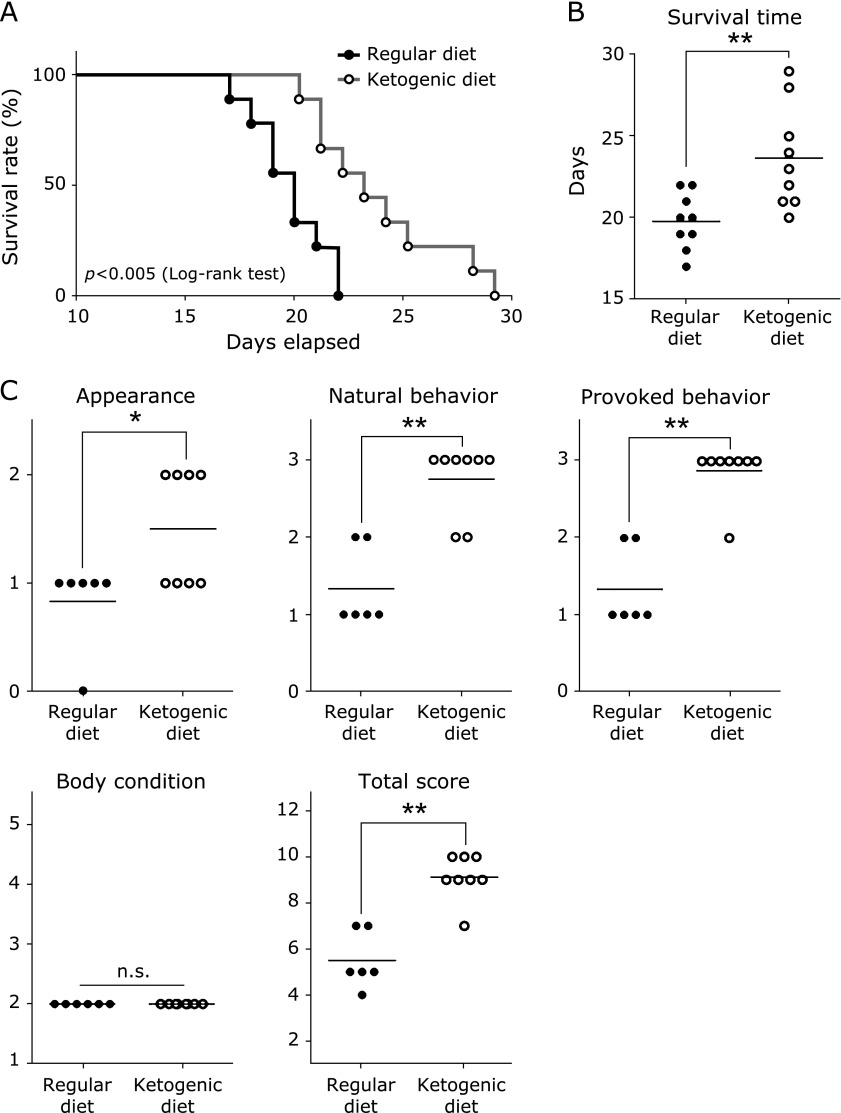
Influence of ketogenic diet on survival time and health condition in mice with peritoneal dissemination. (A) Kaplan-Meier survival curve of colon 26-bearing mice fed a regular diet or ketogenic diet. Log-rank test for survival distribution was performed (*p*<0.005). (B) Survival time in peritoneal dissemination mice. Each point represents the value from one mouse; horizontal bars indicate the mean. Welch’s *t* test was performed. (C) Health condition score of mice with peritoneal dissemination at day 18. Each point represents the value from one mouse; horizontal bars indicate the mean. Statistical significance was determined by Student’s *t* test. *p<0.05, **p<0.01. n.s., not significant. Results are representative of three independent experiments.

We examined the influence of a ketogenic diet on survival time and health conditions in a mouse model of peritoneal dissemination. Mice fed a ketogenic diet had a statistically increased survival time compared with mice fed a regular diet (Fig. 1A). The ketogenic diet group had a significantly increased mean survival time compared with the regular diet group (Fig. 1B). Health condition, appearance, natural behavior, and provoked behavior scores were significantly higher in the ketogenic diet group compared with the regular diet group, whereas body condition scores (score 2) were not different between groups. The total score was higher in the ketogenic diet group compared with the regular diet group (Fig. 1C). Of note, the natural behavior and provoked behavior scores in the ketogenic diet group were very high.

### Ketogenic diet does not inhibit tumor growth but reduces ascites in mice with peritoneal dissemination

**Fig. 2:**
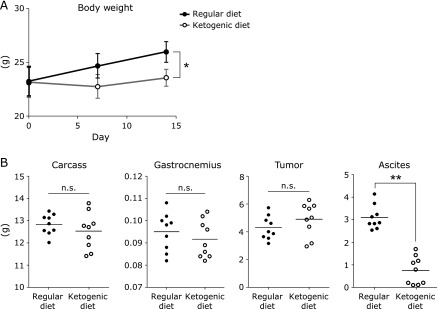
Changes in body weight, ascites, muscle, carcass weight, and tumor weight of peritoneal dissemination mice. Mice inoculated with colon 26 cells were fed a regular diet or ketogenic diet for 15 days. The weight of individual mice was determined weekly. Results shown are the mean ± SD. Carcass, gastrocnemius muscle, tumor weight, and ascites weight were plotted for each group on the day of necropsy. Each point represents the value from one mouse; horizontal bars indicate the mean. Carcass weight was determined by weight of mouse body with all organs removed (eviscerated) except the head. Statistical significances were determined by the Student’s *t* test. *p<0.05, **p<0.01. n.s., not significant. Similar results were obtained in three independent experiments.

To gain insight into how the ketogenic diet affects the survival time in mice with peritoneal dissemination, we performed a necropsy to measure various parameters before the health condition began to decline markedly. The body weight of the ketogenic diet group was significantly lower compared with the regular diet group, but there was no significant difference in the weight of the carcass and gastrocnemius on the day of necropsy (Fig. 2). Remarkably, there was no significant difference in peritoneal tumor weight between the two groups, but mice receiving the regular diet had a large amount of hemorrhagic ascites, whereas mice fed the ketogenic diet had almost no ascites accumulation. These results suggest that the body weight of the regular diet group was increased because of increased ascites accumulation and that the prolongation of survival time in the ketogenic diet group was not due to peritoneal tumor reduction but rather an improvement in ascites accumulation.

### Ketogenic diet increases albumin and total protein in blood and improves anemia in mice with peritoneal dissemination

**Fig. 3:**
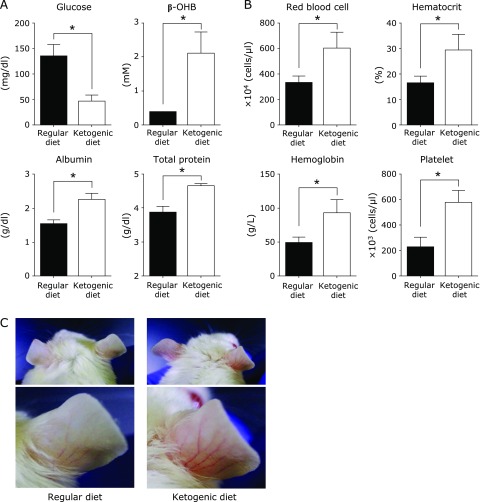
Serum chemistry and hematological tests in the mouse model of peritoneal dissemination. (A) Glucose, albumin, and total protein were measured on day 15. β-OHB was measured immediately after blood collection. Results shown are the mean ± SD. Statistical significance for glucose, albumin, and total protein was determined by the Student’s *t* test and for β-OHB by Welch’s *t* test. (B) Hematological analysis was performed using blood collected in EDTA coated tubes. Results shown are the mean ± SD. Statistical significance was determined by the Student’s *t* test. (C) Representative images of ear complexion and blood vessels on day 15 in mice from each group. Results are representative of three independent experiments. *p<0.01.

We next assessed biochemical markers in the blood to evaluate the effect of a ketogenic diet on mouse nutritional status. Blood glucose levels in the ketogenic diet group were lower than that of the regular diet group and ketone body concentrations in the ketogenic diet group were higher than that in the regular diet group as expected (Fig. 3A). Serum albumin and total protein concentration were also increased in the ketogenic diet group compared with the regular diet group. The difference in serum albumin and total protein concentration was probably not a reflection of nutritional status but rather because of the presence or absence of the leakage of blood components caused by ascites retention as no difference in carcass and muscle weight was observed at necropsy.

Mice fed a regular diet and inoculated with colon 26 cells in the peritoneal cavity showed severe anemia symptoms with a pale complexion in the ears as time proceeded. In contrast, mice fed the ketogenic diet did not show obvious anemia symptoms and hematological assessment revealed that red blood cell, hematocrit, and hemoglobin were higher in the ketogenic diet group than in the regular diet group (Fig. 3B and C). These data clearly indicate that the suppression of hemorrhagic ascites retention was associated with an improvement of anemia.

### Ketogenic diet decreases the production of VEGF-A from tumor cells *in vivo*

**Fig. 4:**
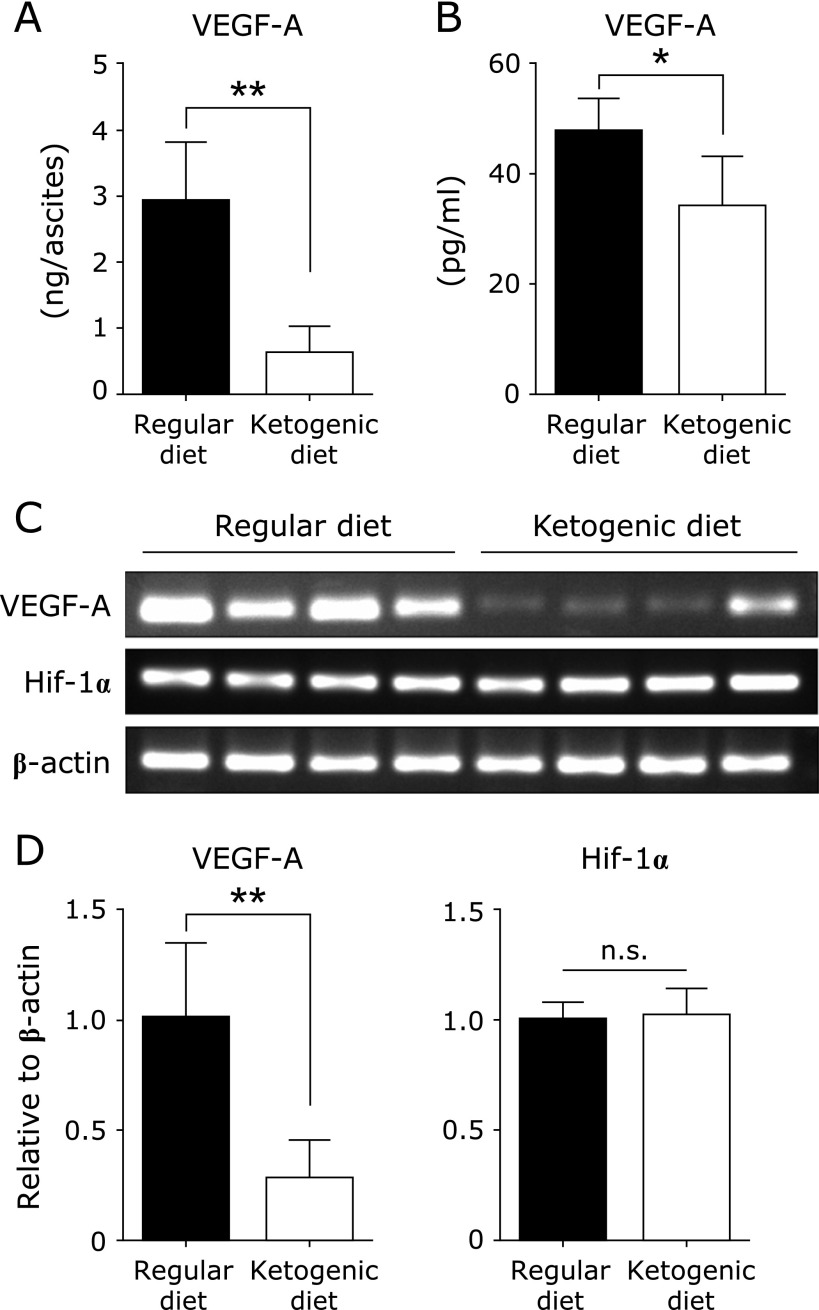
VEGF-A levels in ascites and serum, and semi-quantitative RT-PCR of peritoneal tumor cells. (A) Total VEGF-A content in the ascites from each group. Ascites were centrifuged and supernatants were used for VEGF-A ELISA. (B) VEGF-A in the serum from each group was measured by ELISA. Results shown are the mean ± SD. (C) Optical densities of VEGF-A, Hif1α, and β-actin mRNA levels. Results were analyzed by semi-quantitative RT-RCR. (D) The expressions of VEGF-A and Hif1α were normalized by β-actin. Results shown are the mean ± SD. Statistical significance was determined by the Student’s *t* test. *p<0.05, **p<0.01. n.s., not significant.

Next, we focused on the effect of decreasing ascites fluid retention by ketogenic diet. The formation of ascites involves increased peritoneal microvasculature, endothelial cell permeability, invasion and metastasis of tumor cells, and the anoxic environment of the peritoneal cavity, which are associated with VEGF-A.^(17–20)^ Therefore, we tested whether the ketogenic diet affected the production of VEGF-A, which is involved in the accumulation of ascites.^(21)^ Mice in the ketogenic diet group showed a significant decrease in serum and ascites VEGF-A levels compared with the regular diet group (Fig. 4A and B). In addition, VEGF-A mRNA levels in the peritoneal tumor were low in the ketogenic diet group. The VEGF gene is induced by Hif-1, a transcription factor, and Hif-1 transcriptional activity depends on the expression of the Hif-1α subunit.^(22)^ Therefore, we measured Hif-1α mRNA levels in tumors, but there was no significant difference between the two groups (Fig. 4C and D). Although the mechanism involved is unclear, these data imply that the ketogenic diet reduces ascites accumulation by suppressing VEGF-A production from tumors at the transcription level.

### Ketone bodies do not suppress VEGF-A in tumor cells *in vitro*

**Fig. 5:**
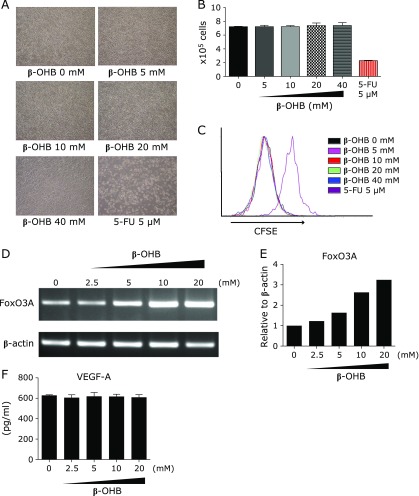
Effects of β-OHB on colon 26 proliferation and VEGF-A production *in vitro*. (A) Representative photomicrographs of colon 26 cells in the absence or presence of β-OHB at the indicated concentrations after 48 h. The photomicrographs were taken at ×40 magnification. (B) Cell counting was performed by WST-8 assay. Results shown are the mean ± SD. (C) Cell proliferation evaluated by CFSE-dilution assay. CFSE-labeled colon 26 cells were treated with different doses of β-OHB. After 48 h, the CFSE fluorescence intensity was quantified by flow cytometry analyses. (D) Optical densities of FoxO3A and β-actin mRNA levels in colon 26 cells at each concentration of β-OHB after 48 h incubation. Results were analyzed by semi-quantitative RT-RCR. (E) The expression of FoxO3A was normalized by β-actin. (F) Colon 26 cells were treated with different doses of β-OHB. After 48 h, VEGF-A in the culture supernatant was measured by ELISA. Results shown are the mean ± SD. 5-FU, 5-Fluorouracil.

To explore the detailed mechanism of the inhibition of VEGF-A production from colon 26 tumor cells by ketogenic diet, we investigated the function of a ketone body, β-OHB, against tumor cells *in vitro*. Recent studies reported that β-OHB upregulated the transcription levels of FoxO3A via chromatin acetylation and FoxO3A suppressed VEGF-A expression by binding to its promoter and deacetylating histone.^(13,23)^ Additionally, FoxO3A interacted with Hif-1α and negatively regulated Hif-1α transcriptional activity.^(24)^ Therefore, we hypothesized that improved ascites accumulation by ketogenic diet was related to the decreased expression of VEGF-A by increasing the expression of FoxO3A by β-OHB. To test this, colon 26 cells were treated with β-OHB at various concentrations and incubated for 48 h. β-OHB at any concentration had no effect on the number or proliferation of tumor cells, similar to the results *in vivo* (Fig. 5A–C). As shown in Fig. 5D and E, the expression of FoxO3A was increased in a β-OHB dose-dependent manner, but unexpectedly, the production of VEGF-A was unchanged at all concentrations tested (Fig. 5F).

## Discussion

In this study, we demonstrated that a ketogenic diet prolonged the survival time and improved ascites accumulation, nutritional markers, and anemia symptoms without suppressing tumor progression in a mouse model of peritoneal dissemination. Recent studies reported that ketogenic diet suppressed tumor progression and ketone bodies directly decreased the viability of tumor cells.^(25,26)^ Here, we found that a ketogenic diet increased the ketone body concentration in the blood of mice with peritoneal dissemination, although there was no change in the tumor weight *in vivo*. Furthermore, β-OHB, a ketone body, did not alter the growth of tumor cells *in vitro*. Other studies reported that ketone bodies promoted the development of human breast cancer cells.^(27)^ Not all tumors are involved in aerobic glycolysis, “the Warburg effect”, and cancer cells utilize both glycolysis and oxidative phosphorylation by mitochondria to satisfy their metabolic requirements.^(28)^ The differences between these reports might be related to different pathways of energy production specific for each cancer cell. Therefore, this suggests that β-OHB had no antitumor activity because the colon 26 cells used in this study might not depend on aerobic glycolysis.

Surprisingly, the mice fed ketogenic diet had markedly reduced ascites accumulation compared with mice in the regular diet group, even in the absence of tumor regression. This suggests a direct effect that extended the survival time of mice with peritoneal dissemination. Additionally, although there was an increase in blood albumin and total protein concentration in mice fed the ketogenic diet, there was no change in skeletal muscle weight. These results suggest that the blood components did not leak into the peritoneal cavity as ascites, but that they improved the nutritional status. Moreover, the concentration of VEGF-A in the blood and ascites, which is associated with ascites retention, was also decreased in the ketogenic diet group. Our study is the first to report that the survival time of tumor bearing mice was prolonged without suppression of tumor progression, and that the accumulation of ascites was suppressed via the inhibition of VEGF-A production by a ketogenic diet. However, *in vitro*, we could not detect any difference in VEGF-A levels in the presence of β-OHB. These observations indicate that the inhibition of VEGF-A from tumor cells *in vivo* was not a direct effect of the ketone body. Indeed, mice fed the ketogenic diet had a low glucose concentration in the blood; however, we consider the low glucose concentration was not associated with the reduction of ascites retention for the following reasons. First, the low glucose concentration decreased the number of tumor cells *in vitro*, which did not reflect the results *in vivo* (data not shown). Second, it is generally accepted that cancer cells increase VEGF-A expression for angiogenesis to escape metabolic deprivation under low glucose conditions.^(29–31)^ Thus, further studies are required to investigate why the ketogenic diet reduced ascites accumulation and to provide a more detailed mechanism related to the inhibition of VGEF-A production from tumor cells.

In the present study, mice fed the ketogenic diet had an improved health score, indicating that blood components did not leak as ascites. Notably, the scores for natural behavior and provoked behavior in the ketogenic diet group were approximately 3, similar to that of normal mice, even though the other scores were reduced and there was no difference in the muscle weight between the two groups. This suggests that the ketogenic diet improved motor performance in the mouse peritoneal dissemination model. Recent studies reported that a ketogenic diet improved motor performance in a mouse model of Alzheimer’s disease without affecting the level of β-amyloid, which plays a crucial role in the pathogenesis of the disease.^(32,33)^ Interestingly, in a study by Brownlow *et al.*,^(32)^ the motor performance of Alzheimer’s disease mice and normal mice fed a ketogenic diet was increased. These findings suggest that a ketogenic diet increases muscle motor performance regardless of treatment for the underlying cause of disease. Because a ketogenic diet increases ATP levels, the capacity for biological energy, and transcription of enzymes in energy-producing pathways, it is also likely that levels of metabolic intermediates may be altered.^(34)^ Type I muscle fibers, slow muscle fibers, are mitochondria-rich and mainly use β-oxidation for energy production, which provides a stable and long-lasting supply of ATP, compared with type II muscle fibers, fast muscle fibers.^(35)^ Based on these reports and our observations, the improvement of motor performance by ketogenic diet may be related to enhanced efficient ATP production by supplying fat and ketone bodies to muscle, especially type I muscle fibers.

Although small scale studies with limited evidence have been reported, clinical trials using a ketogenic diet for cancer patients reported it was safe and effective in general.^(36–38)^ Indeed, a ketogenic diet was shown to be safe in patients with epilepsy and congenital metabolic disease such as glucose transporter 1 deficiency. Additionally, we observed no adverse effects in mice attributable to the ketogenic diet. However, high-quality evidence on the effect of a ketogenic diet is lacking in cancer patients. As mentioned above, there is a possibility that the antitumor effect of a ketogenic diet is hard to detect in clinical trials because of differences in the metabolic mechanisms for different cancers. Many reports of animal experiments focus on the antitumor effect of a ketogenic diet, but we would like to emphasize the ascites and motor performance improving effect. Based on our observations, a ketogenic diet might improve ascites retention and reduce abdominal discomfort associated with peritoneal dissemination. Moreover, it was reported that a ketogenic diet reduced tumor levels of lactic acid, a potential cause of tiredness.^(39)^ Therefore, a ketogenic diet might reduce fatigue by inhibiting the synthesis of lactic acid and promoting efficient ATP production in muscle. Although a ketogenic diet may not be effective for bowel obstructions because it does not shrink tumors, it might contribute to improving quality of life (QOL) in patients with peritoneal dissemination.

In summary, a ketogenic diet prolonged the survival time, and improved motor performance and ascites accumulation without suppressing tumor growth in a mouse model of peritoneal dissemination. The amelioration of ascites might be related to the attenuation of VEGF-A in the tumor, but the detailed mechanism is unclear. A ketogenic diet may enhance efficient ATP production in muscle. Clinically, a ketogenic diet might be one approach to improve the QOL in peritoneal dissemination patients.

## Author Contributions

E. Kasumi performed all experiments and wrote the manuscript. N. Sato supervised the overall research project.

## Figures and Tables

**Table 1:** Composition of the regular and ketogenic diets used in this study

Ingredients	Regular diet	Ketogenic diet
	(g/100 g diet)	
Casein	20.3	14.4
Soybean Oil	7.0	72.4
Dextrin	63.2	3.7
AIN-93G mineral mix	3.5	3.5
AIN-93 vitamin mix	1.0	1.0
Cellulose	5.0	5.0

The regular diet was based on the composition of AIN-93G. The ketogenic diet consisted of powdered oil obtained by freeze-drying an emulsion of casein and soybean oil that could be eaten by animals. All ingredients were purchased from Oriental Yeast Co., Ltd. (Tokyo, Japan).

## Abbreviations

ATP: adenosine triphosphate
β-OHB: β-hydroxybutyrate
CFSE: carboxyfluorescein diacetate succinimidyl ester
FoxO3A: forkhead box O 3A
Hif: hypoxia-inducible factor
PBS: phosphate-buffered saline
QOL: quality of life
VEGF-A: vascular endothelial growth factor A
